# Effects of mHealth-Based Lifestyle Interventions on Gestational Diabetes Mellitus in Pregnant Women With Overweight and Obesity: Systematic Review and Meta-Analysis

**DOI:** 10.2196/49373

**Published:** 2024-01-17

**Authors:** Yirong He, Chuanya Huang, Qiuyang He, Shujuan Liao, Biru Luo

**Affiliations:** 1 Department of Nursing West China Second University Hospital Sichuan University Chengdu, Sichuan China; 2 Key Laboratory of Birth Defects and Related Diseases of Women and Children Sichuan University Ministry of Education Chengdu, Sichuan China; 3 Department of Obstetric Nursing West China Second University Hospital Sichuan University Chengdu, Sichuan China

**Keywords:** mobile health, mHealth, lifestyle intervention, gestational diabetes mellitus, meta-analysis, mobile phone

## Abstract

**Background:**

The increasing incidence of gestational diabetes mellitus (GDM) is a global health problem that is more likely to occur in pregnant women with overweight or obesity. Adhering to a healthy lifestyle is associated with a reduced risk of GDM. With the development of IT, mobile health (mHealth) interventions have become widely available in health care. However, there are no definitive conclusions on the effectiveness of mHealth-based lifestyle interventions in preventing GDM.

**Objective:**

This study aims to evaluate the impact of mHealth-based lifestyle interventions on GDM and other pregnancy outcomes in pregnant women with overweight or obesity.

**Methods:**

A systematic literature search was conducted in 5 English databases (MEDLINE, Embase, Web of Science, CENTRAL, and CINAHL) and 4 Chinese databases (CBM, CNKI, Vip, and Wanfang) to identify randomized controlled trials (RCTs) on the effectiveness of mHealth-based interventions for GDM from inception to January 10, 2023. In total, 2 authors independently screened the studies and extracted the data. The quality of the included studies was examined using the Cochrane risk-of-bias tool. Data synthesis was conducted using Review Manager (version 5.4; The Cochrane Collaboration).

**Results:**

A total of 16 RCTs with 7351 participants were included in this study. The included studies were published between 2014 and 2021 and were conducted in China, the United States, Australia, New Zealand, the United Kingdom, Ireland, and Norway. The sample sizes of the studies ranged from 75 to 2202, and the duration of the mHealth-based lifestyle interventions ranged from 4 to 28 weeks. Compared with usual care, mHealth-based lifestyle interventions significantly reduced the incidence of GDM (odds ratio [OR] 0.74, 95% CI 0.56-0.96; *P*=.03; *I*^2^=65%), preterm birth (OR 0.65, 95% CI 0.48-0.87; *P*=.004; *I*^2^=25%), macrosomia (OR 0.59, 95% CI 0.40-0.87; *P*=.008; *I*^2^=59%), and gestational weight gain (mean difference=−1.12 kg, 95% CI −1.44 to −0.80; *P*<.001; *I*^2^=43%). The subgroup analysis showed that interventions delivered via apps (OR 0.55, 95% CI 0.37-0.83; *P*=.004; *I*^2^=44%), provided by obstetricians (OR 0.69, 95% CI 0.51-0.93; *P*=.02; *I*^2^=60%), and targeted at Asian populations (OR 0.44, 95% CI 0.34-0.58; *P*<.001; *I*^2^=0%) and that used the International Association of Diabetes and Pregnancy Study Groups diagnostic criteria (OR 0.58, 95% CI 0.39-0.86; *P*=.007; *I*^2^=69%) showed a statistically significant reduction in the risk of GDM.

**Conclusions:**

mHealth-based lifestyle interventions had a favorable impact on the prevention of GDM in pregnant women with overweight and obesity. Future studies need to further explore the potential of mHealth-based interventions for GDM through better design and more rigorous large-scale RCTs.

**Trial Registration:**

PROSPERO International Prospective Register of Systematic Reviews CRD42021286995; https://www.crd.york.ac.uk/prospero/display_record.php?RecordID=286995

## Introduction

### Background

Gestational diabetes mellitus (GDM) is defined as a carbohydrate intolerance of varying severity with onset or first recognition during pregnancy [1]. GDM is one of the most common obstetric complications, with the prevalence varying from 7.5% to 27% in different areas [2]. GDM is associated with substantial adverse pregnancy outcomes such as neonatal hypoglycemia and macrosomia as well as long-term metabolic risk in pregnant women and their offspring [3]. Risk factors for GDM include age, ethnicity, overweight or obesity, and family history of diabetes [4]. As obesity becomes a global epidemic, perinatal overweight and obesity are also a growing concern [5]. In recent decades, the rates of overweight and obesity among women of reproductive age have increased dramatically [6]. In the United States, 60% of women are overweight or obese during pregnancy compared with 30% in Europe and 10% in Asia [7]. In pregnant women with overweight and obesity, it is estimated that the risk of GDM is more than twice that of other pregnant women [8]. Unhealthy lifestyle behaviors are a critical factor that affects overweight and obesity during the perinatal period [9]. Numerous randomized controlled trials (RCTs) have attempted to reduce the incidence of GDM through diet [10], physical activity [11], or combination interventions [12]. However, when lifestyle interventions are provided in a personalized way, they are commonly expensive and may lack scalability from the perspective of public health [13]. Therefore, there is an urgent need for effective real-world solutions that address the demand of pregnant women seeking personalized support, information, and guidance to help reduce the risk of GDM.

As IT advances and more people use electronic devices, mobile health (mHealth) has developed rapidly [14]. mHealth is commonly defined as health care services provided by health care professionals using telecommunications technology [15]. mHealth has been applied in many areas of perinatal health care, with medical staff providing health care services to pregnant women through multimedia methods such as mobile apps, software, SMS text messages, email, web-based diaries, and integrated systems combining various components of digital communications technologies [16]. Studies have shown that mHealth care can reduce gestational weight gain (GWG); improve pregnant women’s health behaviors; and reduce the number of medical visits, thereby decreasing financial burden [13]. However, the impact of mHealth interventions on pregnancy outcomes in women with overweight or obesity is uncertain. More and more systematic reviews have found an effect of lifestyle interventions based on mHealth technology on diabetes prevention among adults with overweight and obesity [17,18]; however, little is known about their effectiveness in the perinatal population. Several previous reviews have attempted to synthesize the results of mHealth-based lifestyle programs for pregnant women, but none have evaluated the quantitative effects of these programs [19,20]. There is still no consensus on the impact of mHealth lifestyle interventions on preventing GDM and other pregnancy outcomes in women with overweight or obesity.

### Objectives

Therefore, we conducted a systematic review and meta-analysis of RCTs to summarize mHealth interventions delivered in different ways and assess the effectiveness of mHealth-based lifestyle interventions in reducing the risk of GDM.

## Methods

This systematic review and meta-analysis follows the PRISMA (Preferred Reporting Items for Systematic Reviews and Meta-Analyses) guidelines [21] and is presented in Multimedia Appendix 1. It was registered in PROSPERO on November 18, 2021, with registration number CRD42021286995.

### Search Strategy

A systematic literature search was conducted in 5 English databases (MEDLINE, Embase, Web of Science, CENTRAL, and CINAHL) and 4 Chinese databases (CBM, CNKI, Vip, and Wanfang) for studies published in English and Chinese. A systematic search was conducted combining Medical Subject Headings (MeSH) and free-text terms, including *overweight* (MeSH) OR *obesity* (MeSH); pregnan* OR *prenatal* OR *antenatal* OR *maternal* OR *gestational*; *gestational diabetes mellitus* OR *gestational diabetes* OR *GDM* OR *T2DM* OR *impaired fasting glucose* OR *impaired glucose tolerance*; *telemedicine* (MeSH) OR *telerehabilitation* (MeSH) OR *telecommunications* (MeSH) OR *electronic health* OR *eHealth* OR *ehealth* OR *mHealth* OR *mobile health* OR *telecare* OR *eHealthcare* OR *mcare* OR telemonitor* OR telerehab* OR *telemanagement* OR *mobile communication* OR *remote consult* OR mobile technolog* OR mobile devic* OR mobile app* OR *internet* (MeSH) OR web* OR *online* OR *smartphone* (MeSH) OR *telephone* (MeSH) OR *cell phone* (MeSH) OR *cellular phone* (MeSH) OR *mobile phone* OR messag* OR *SMS*. The searches were unlimited by time up to January 10, 2023, and were limited to RCTs. The full details of the search strategy for each database are provided in Multimedia Appendix 2. We complemented this strategy by manually searching the reference lists of included studies and related reviews.

### Inclusion and Exclusion Criteria

In total, 2 researchers (YH and CH) independently screened the titles and abstracts and selected the studies in accordance with the eligibility criteria. Any disagreement was resolved through discussion with a third researcher (QH). Studies were included if they met the following inclusion criteria: (1) pregnant women with overweight (BMI ≥25 kg/m^2^) or obesity (BMI ≥30 kg/m^2^; population); (2) mHealth interventions including pregnancy nutrition, physical activity, weight management, and health behavior education delivered via the internet, websites, telephone, app, SMS text message, email, or other types of information and communications technologies (intervention); (3) usual care, routine care, conventional care, or standard care without mHealth (comparison); (4) incidence of GDM, postpartum hemorrhage, preterm birth, cesarean delivery, pregnancy-induced hypertension, macrosomia, neonatal gestational age, and GWG (outcome); (5) RCTs (study design); and (6) English or Chinese (language). There were no restrictions regarding the year of publication. We excluded studies that (1) included women with either type 1 or type 2 diabetes mellitus before pregnancy or with existing GDM; (2) contained incomplete data; (3) lacked data related to GDM; or (4) were study protocols, comments, editorials, and conference abstracts.

### Study Selection and Data Extraction

The reference management program EndNote X9 (Clarivate Analytics) was used for data management. The studies were imported into EndNote after an extensive database search. After removing duplicates, 2 authors independently reviewed the titles and abstracts according to the eligibility criteria. Disagreements were resolved through discussion or consultation with a third researcher. Data were extracted by an independent researcher (YH) using the predesigned data collection forms, and the extracted data were verified by a second researcher (CH). Disagreements were resolved through consensus. The extracted data included the authors, year, country, study design, sample size, participant characteristics, intervention, control, GDM criteria, and outcomes.

### Quality Assessment

In total, 2 researchers (YH and CH) independently assessed the studies’ risk of bias in accordance with the *Cochrane Handbook for Systematic Reviews of Interventions*. This tool consists of 6 items: selection bias, performance bias, detection bias, attrition bias, reporting bias, and other biases. Each item was judged as “low risk,” “high risk,” or “unclear risk.” A third researcher (QH) was available if there was a difference in opinion in assessing the risk of bias.

### Data Synthesis and Analysis

Statistical analyses were conducted using Review Manager (version 5.4; The Cochrane Collaboration). The overall effect difference was considered statistically significant if the 2-tailed *P* value was <.05. Continuous variables were presented using the mean difference, and dichotomous variables were described using the odds ratio (OR) with a 95% CI. Heterogeneity was assessed using the Cochran *Q* test and the *I*^2^ statistic. We considered <25%, 25% to 50%, 50% to 75%, and >75% as low, moderate, high, and severe heterogeneity between the studies, respectively. If *I*^2^≤50% and the *P* value was >.10, a fixed-effects model was considered; otherwise, a random-effects model was used. The sources of heterogeneity were explored using subgroup analysis. A funnel plot was constructed to check for potential publication bias.

## Results

### Study Selection

**Figure 1:**
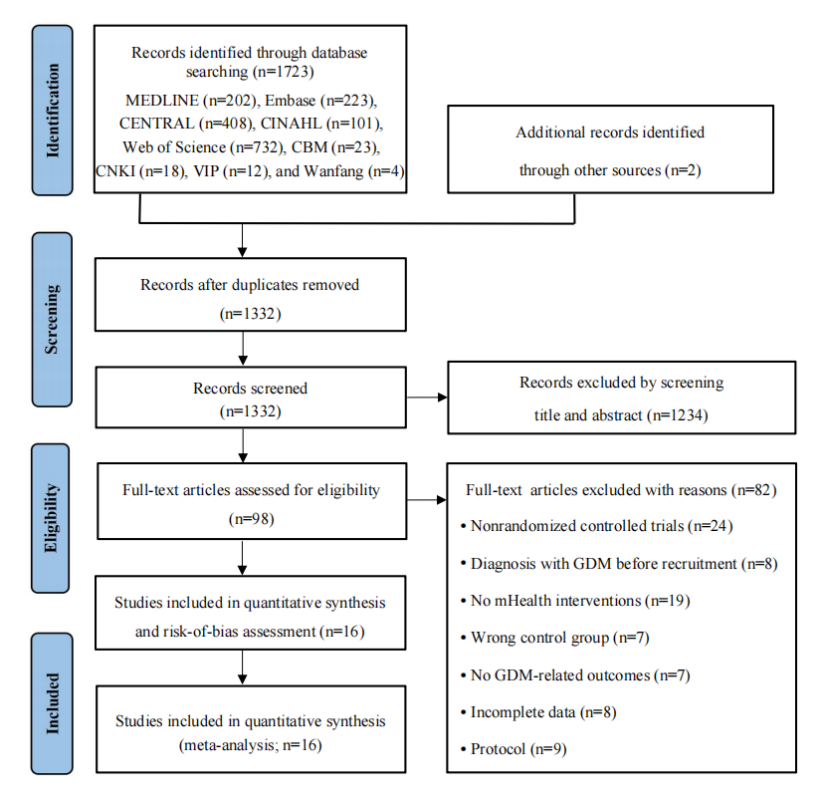
Flowchart of study selection. GDM: gestational diabetes mellitus; mHealth: mobile health.

Figure 1 shows a PRISMA flowchart of the study selection process. A total of 1725 records were retrieved from 9 electronic databases. After removing duplicates (393/1725, 22.78% of the studies), 1332 studies were included for screening. Of these 1332 studies, we then excluded 1234 (92.64%) based on the relevance of the abstract and title, and the remaining 98 (7.36%) studies were assessed for eligibility. After full-text review, 84% (82/98) of the studies were excluded for the reasons outlined in Figure 1. Finally, 16 studies were included in the review and meta-analysis.

### Characteristics of the Included Studies

#### Study Characteristics

The characteristics of the included studies are shown in Table 1. The 16 studies included in this review and meta-analysis were RCTs, 4 (25%) of which were multicenter RCTs. The included studies were published between 2014 and 2021 and were conducted in China (8/16, 50%) [22-29], the United States (2/16, 12%) [30,31], Australia (2/16, 12%) [32,33], New Zealand (1/16, 6%) [34], the United Kingdom (1/16, 6%) [35], Ireland (1/16, 6%) [36], and Norway (1/16, 6%) [37]. A total of 7351 participants were included in the studies, and the sample sizes varied from 74 [34] to 2153 [32]. In total, 38% (6/16) of the studies included pregnant women with obesity [23,24,26,28,33,35], and 62% (10/16) included both pregnant women with overweight and pregnant women with obesity [22,25,27,29,30,31,32,34,36, 37]. A total of 81% (13/16) of the articles reported diagnostic criteria for GDM. The incidence of GDM was determined by screening pregnant women using an oral glucose tolerance test. The diagnostic criteria for GDM were inconsistent among the included studies. In total, 56% (9/16) of the RCTs used the International Association of Diabetes and Pregnancy Study Groups (IADPSG) criteria (2010) [22,23,25,27-29,34-36], 12% (2/16) used the World Health Organization 2013 criteria [33,37], 6% (1/16) used the Australasian Diabetes in Pregnancy Society criteria [32], and 6% (1/16) of the RCTs used the Carpenter-Coustan criteria [30]. All the RCTs (16/16, 100%) reported more than 1 outcome.

**Table 1:** Characteristics of the included studies.

Author, year, and country	Study design	Sample size	Participant characteristics	Intervention	Control	GDM^a^ criteria	Outcomes
Dodd et al [32], 2014, Australia	Multicenter RCT^b^	2153; IG^c^: 1080; CG^d^: 1073 (1:1)	Women with a singleton pregnancy between 10 and 20 wk of gestation and a BMI of ≥25 kg/m^2^	Phone, in person	Standard care	ADPSC^e^	LGA^f^, macrosomia, PIH^g^, pre-eclampsia, GDM, PTB^h^, CS^i^, and PPH^j^
Sagedal et al [37], 2015, Norway	RCT	591; IG: 296; CG: 295 (1:1)	Women with a singleton pregnancy at ≤20 wk of gestation who had a prepregnancy BMI of ≥25 kg/m^2^	Phone, website, in person	Standard care	WHO^k^	GWG^l^, GDM, LGA, SGA^m^, pre-eclampsia, PTB, PPH, and NW^n^
Seneviratne et al [34], 2015, New Zealand	RCT	74; IG: 37; CG: 37 (1:1)	Women aged 18-40 y with a BMI of ≥25 kg/m^2^ and a singleton pregnancy of <20 wk of gestation	Software, device, home-based, in person	Usual care	IADPSG^o^	NW, hypoglycemia, GWG, GDM, PIH, PTB, CS, PPH, MVPA^p^, and pre-eclampsia
Poston et al [35], 2015, United Kingdom	Multicenter RCT	1280; IG: 629; CG: 651 (1:1)	Women aged ≥16 y with a BMI of ≥30 kg/m² and a singleton pregnancy between 15 and 18 wk plus 6 d of gestation	Phone, email, DVD, pedometer, logbook, in person	Standard care	IADPSG	GDM, FBG^q^, pre-eclampsia, CS, PPH, GWG, HOMA-IR^r^, GL^s^, GI^t^, fat, MVPA, LGA, and NW
Simmons et al [33], 2017, Australia	Multicenter RCT	192; IG: 92; CG: 100 (1:1)	Women with a BMI of >29 kg/m^2^, ≤19 (–6 to , 6) d of gestation, and a singleton pregnancy	Email, phone, pedometer, device, in person	Usual care	WHO	GDM, FBG, HOMA-IR, insulin, NW, LGA, SGA, MVPA, and sugar intake
Chen [22], 2017, China	RCT	160; IG: 80; CG: 80 (1:1)	Women with a prepregnancy BMI of ≥24 kg/m^2^ and a singleton pregnancy between 8 and 12 wk of gestation	Mobile apps, SMS text messages, in person	Usual care	IADPSG	BMI, GDM, CS, and NW
Kennelly et al [36], 2018, Ireland	RCT	498; IG: 241; CG: 257 (1:1)	Singleton pregnant women between 10 and 15 wk of gestation with a BMI between 25.0 and 39.9 kg/m^2^	Mobile app, email, in person	Usual care	IADPSG	GDM, GWG, GL, GI, PIH, CS, HOMA-IR, NW, LGA, and SGA
Li [23], 2018, China	RCT	1000; IG: 500; CG: 500 (1:1)	Women with a BMI of ≥28 kg/m^2^, aged >18 y, and with a singleton pregnancy between 8 and 12 wk of gestation	Phone, in person	Usual care	IADPSG	GDM, GWG, NW, and macrosomia
Tang et al [24], 2019, China	RCT	136; IG: 68; CG: 68 (1:1)	Pregnant women with a BMI of ≥28 kg/m^2^ aged >18 y	Mobile app, software	Standard care	N/A^u^	Macrosomia, CS, hypoglycemia, and GDM
Ferrara et al [30], 2020, United States	Multicenter RCT	389; IG: 195; CG: 194 (1:1)	Women at 8-15 wk of gestation with singletons, with a prepregnancy BMI of 25-40 kg/m², and aged ≥18 y	Phone, device, in person	Usual care	Carpenter and Coustan criteria	GWG, caloric intake, MVPA, FBG, HOMA-IR, GDM, PIH, pre-eclampsia, CS, NW, and macrosomia
Cao [25], 2020, China	RCT	96; IG: 48; CG: 48 (1:1)	Women aged 22-38 y with a BMI of ≥24 kg/m^2^ and a singleton pregnancy between 8 and 12 wk of gestation	Mobile apps, SMS text messages, in person	Usual care	IADPSG	FBG, GDM, BMI, CS, NW, and LGA
Wu and Guang [26], 2020, China	RCT	140; IG: 70; CG: 70 (1:1)	Pregnant women with a BMI of ≥28 kg/m^2^ aged >18 y	Mobile app, in person	Usual care	N/A	BMI, NW, GDM, PIH, CS, PTB, macrosomia, and PPH
Liu et al [31], 2021, United States	RCT	217; IG: 112; CG: 105 (1:1)	Pregnant women aged 18-44 y with a gestational age of ≤16 wk and a prepregnancy BMI of ≥25 kg/m^2^	Phone, software, SMS text messages, in person	Standard care	N/A	GWG, PTB, LBW^v^, macrosomia, SGA, GDM, PIH, CS, and NW
Zhou et al [27], 2021, China	RCT	104; IG: 52; CG: 52 (1:1)	Singleton pregnant women with a BMI of ≥24 kg/m^2^ and aged >18 y	Mobile app, in person	Usual care	IADPSG	FBG, 2-hour BG^w^, GDM, CS, PTB, and macrosomia
Kang and Sung [28], 2021, China	RCT	106; IG: 53; CG: 53 (1:1)	Women with a BMI of ≥28 kg/m^2^, aged >18 y, and with a singleton pregnancy between 12 and 20 wk of gestation	Mobile app, in person	Usual care	IADPSG	GDM, FBG, 2-hour BG, HbA_1c_^x^, CS, PTB, and macrosomia
Ding et al [29], 2021, China	RCT	215; IG: 104; CG: 111 (1:1)	Pregnant women with a BMI of ≥24 kg/m^2^ at the onset of pregnancy, aged <35 y, and at <12 wk of gestation	Mobile app, in person	Usual care	IADPSG	Energy intake, GDM, FBG, GWG, CS, PTB, PIH, pre-eclampsia, PPH, NW, and macrosomia

^a^GDM: gestational diabetes mellitus.

^b^RCT: randomized controlled trial.

^c^IG: intervention group.

^d^CG: control group.

^e^ADPSC: Australasian Diabetes in Pregnancy Society criteria.

^f^LGA: large-for-gestational-age infant.

^g^PIH: pregnancy-induced hypertension.

^h^PTB: preterm birth.

^i^CS: cesarean section.

^j^PPH: postpartum hemorrhage.

^k^WHO: World Health Organization.

^l^GWG: gestational weight gain.

^m^SGA: small-for-gestational-age infant.

^n^NW: neonatal weight.

^o^IADPSG: International Association of Diabetes and Pregnancy Study Groups.

^p^MVPA: moderate-to-vigorous physical activity.

^q^FBG: fasting blood glucose.

^r^HOMA-IR: homeostatic model assessment of insulin resistance.

^s^GL: glycemic load.

^t^GI: glycemic index.

^u^N/A: not applicable.

^v^LBW: low birth weight.

^w^BG: blood glucose.

^x^HbA_1c_: glycated hemoglobin.

#### Characteristics of the mHealth Interventions

The details of the mHealth interventions are presented in Multimedia Appendix 3 [22-37]. The interventions were divided into 3 groups: exercise only, diet only, and mixed interventions. Regarding the delivery mode, 12% (2/16) of the studies [23,32] provided mHealth interventions through phone counseling, 25% (4/16) of the studies [26-29] only provided the interventions through mobile apps, and 62% (10/16) of the studies [22,24,25,30,31,33-37] adopted a combination of methods. The duration of the mHealth interventions ranged from 4 to 28 weeks. The mHealth interventions were delivered by various types of personnel, including a single provider in 12% (2/16) of the studies [29,32] and multidisciplinary prenatal care providers in 88% (14/16) of the studies [22-28,30,31,33-37].

#### Characteristics of the Comparators

Most studies (14/16, 88%) briefly described the control group, which generally included regular maternity visits and education on diet and exercise during pregnancy. In the control groups, all studies provided standard or usual care for participants, which was based on the different countries’ perinatal practices and local hospital guidelines.

### Risk of Bias

**Figure 2:**
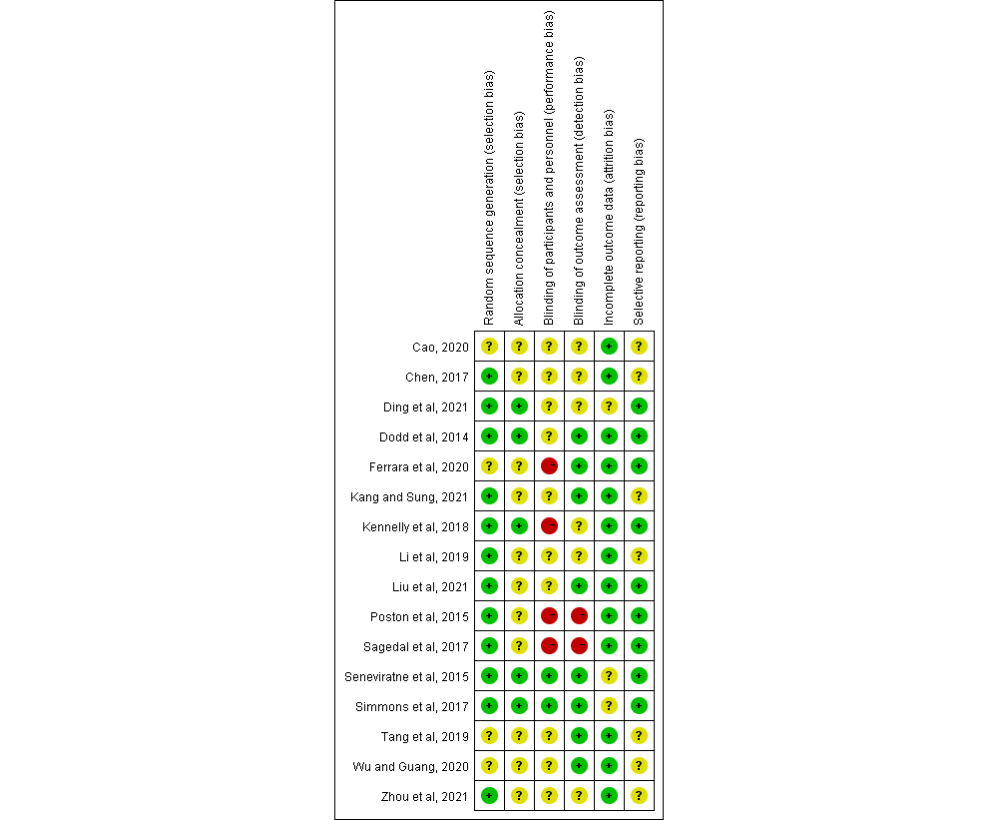
Results of the risk-of-bias assessment of the included studies [22-37].

The Cochrane risk-of-bias tool was used to assess the risk of bias in each study, and the results are shown in Figure 2 [22-37]. A total of 75% (12/16) of the studies described the details of the randomization scheme, and the risk of random sequence generation was low. Only 31% (5/16) of the studies reported allocation concealment, with a low risk of selection bias. Blinding of participants or staff was difficult because of the nature of the mHealth interventions; thus, 25% (4/16) of the studies, which did not blind the participants or staff, were rated as having a high risk of bias, and 62% (10/16) of the studies were rated as unclear regarding the risk of bias. A total of 12% (2/16) of the studies did not report the outcome assessment and were rated as having a high risk of detection bias. In total, 81% (13/16) of the included studies described details of participant dropout and had a low risk of bias. A total of 56% (9/16) of the studies followed preregistered protocols for analysis and outcome reporting, and the remaining 44% (7/16) did not provide information on published protocols or registrations and had an unclear risk of reporting bias. The funnel plot showed no publication bias in any of the included studies (Multimedia Appendix 4).

### Meta-Analysis Results

#### Primary Outcome: the Incidence of GDM

**Figure 3:**
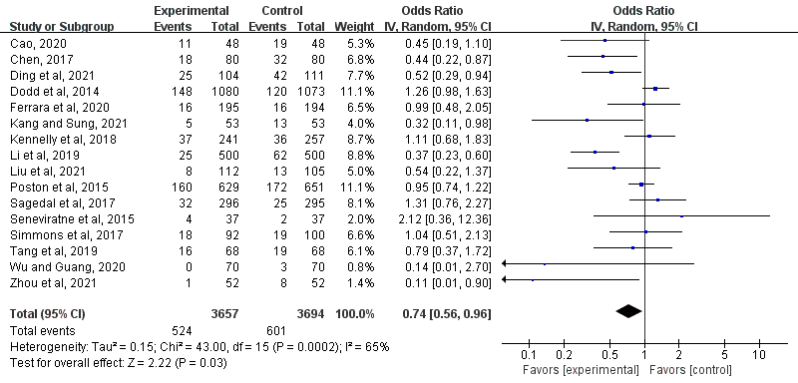
Forest plot of the effect of mobile health interventions on gestational diabetes mellitus [22-37]. IV: inverse variance.

The pooled analysis of the 16 RCTs with 7351 participants showed that the mHealth interventions significantly decreased the incidence of GDM in women with overweight and obesity (OR 0.74, 95% CI 0.56-0.96; *P*=.03; *I*^2^=65%; Figure 3 [22-37]).

Table 2 provides the results of the subgroup analyses, and the forest plots are presented in Multimedia Appendix 5 [22-37]. The subgroup analysis conducted based on the different interventions showed that diet (OR 0.61, 95% CI 0.29-1.28; *P*=.19; *I*^2^=61%), exercise (OR 1.11, 95% CI 0.57-2.17; *P*=.76; *I*^2^=0%), or a combination of the 2 (OR 0.71, 95% CI 0.51-0.99; *P*=.05; *I*^2^=74%) were not statistically significantly associated with reduced risk of GDM. When compared with the control groups, app-based mHealth interventions were significantly effective in reducing GDM (OR 0.55, 95% CI 0.37-0.83; *P*=.004; *I*^2^=44%). Another subgroup analysis conducted based on different providers indicated that interventions provided by obstetricians and nurses were effective in reducing GDM (OR 0.69, 95% CI 0.51-0.93; *P*=.02; *I*^2^=60%). Interventions targeted at Asian populations (OR 0.44, 95% CI 0.34-0.58; *P*<.001; *I*^2^=0%) and using the IADPSG as a diagnostic criterion for GDM showed a reduction in GDM compared with the control groups (OR 0.58, 95% CI 0.39-0.86; *P*=.007; *I*^2^=69%).

**Table 2:** Subgroup analyses of the included studies (n=16).

Subgroup analysis			References		Studies, n (%)		Sample size		Effect estimates (95% CI)		*P* value		*I*^2^ (%)
**Intervention**													
	Diet	Tang et al [24] Zhou et al [27] Kang and Sung [28] Kennelly et al [36]		4 (25)		844		0.61 (0.29-1.28)		.19		61	
	Exercise	Ferrara et al [30] Seneviratne et al [34]		2 (12)		463		1.11 (0.57-2.17)		.76		0	
	Both	Chen [22] Li [23] Cao [25] Wu and Guang [26] Ding et al [29] Liu et al [31] Dodd et al [32] Simmons et al [33] Poston et al [35] Sagedal et al [37]		10 (62)		6044		0.71 (0.51-0.99)		.05		74	
**mHealth^a^ technology**													
	Phone	Ferrara et al [30] Simmons et al [33] Poston et al [35] Sagedal et al [37] Liu et al [31] Dodd et al [32]		6 (38)		4822		1.08 (0.92-1.26)		.37		1	
	App	Chen [22] Tang et al [24] Cao [25] Wu and Guang [26] Zhou et al [27] Kang and Sung [28] Ding et al [29] Kennelly et al [36]		8 (50)		1455		0.55 (0.37-0.83)		.004		44	
	Computer	Li [23] Seneviratne et al [34]		2 (12)		1074		0.72 (0.14-3.74)		.69		71	
**Provider**													
	Dietitian	Ding et al [29] Dodd et al [32]		2 (12)		2368		0.84 (0.36-2.00)		.70		86	
	Exercise physiologists	Seneviratne et al [34]		1 (6)		74		2.12 (0.36-12.36)		.40		N/A^b^	
	Obstetricians	Chen [22] Li [23] Tang et al [24] Cao [25] Wu and Guang [26] Zhou et al [27] Kang and Sung [28] Ferrara et al [30] Liu et al [31] Simmons et al [33] Poston et al [35] Kennelly et al [36] Sagedal et al [37]		13 (81)		4909		0.69 (0.51-0.93)		.02		60	
**Duration**													
	Short term	Zhou et al [27] Liu et al [31] Poston et al [35]		3 (19)		1601		0.60 (0.26-1.39)		.24		61	
	Medium to long term	Chen [22] Li [23] Wu and Guang [26] Kang and Sung [28] Ding et al [29] Ferrara et al [30] Dodd et al [32] Simmons et al [33] Seneviratne et al [34] Kennelly et al [36] Sagedal et al [37]		11 (69)		5518		0.77 (0.53-1.10)		.15		72	
	Long term	Tang et al [24] Cao [25]		2 (12)		232		0.62 (0.35-1.12)		.11		0	
**Ethnicity**													
	Asian	Chen [22] Li [23] Tang et al [24] Cao [25] Wu and Guang [26] Zhou et al [27] Kang and Sung [28] Ding et al [29]		8 (50)		1957		0.44 (0.34-0.58)		<.001		0	
	White	Ferrara et al [30] Liu et al [31] Dodd et al [32] Simmons et al [33] Seneviratne et al [34] Poston et al [35] Kennelly et al [36] Sagedal et al [37]		8 (50)		5394		1.09 (0.93-1.26)		.29		0	
**GDM^c^ diagnostic criteria**													
	IADPSG^d^	Chen [22] Li [23] Cao [25] Zhou et al [27] Kang and Sung [28] Ding et al [29] Seneviratne et al [34] Poston et al [35] Kennelly et al [36]		9 (56)		3533		0.58 (0.39-0.86)		.007		69	
	WHO^e^	Simmons et al [33] Sagedal et al [37]		2 (12)		783		1.20 (0.78-1.86)		.41		0	
	ADPSC^f^	Dodd et al [32]		1 (6)		2153		1.26 (0.98-1.63)		.08		N/A	
	Carpenter-Coustan criteria	Ferrara et al [30]		1 (6)		389		0.99 (0.48-2.05)		.99		N/A	

^a^mHealth: mobile health.

^b^N/A: not applicable.

^c^GDM: gestational diabetes mellitus.

^d^IADPSG: International Association of Diabetes and Pregnancy Study Groups.

^e^WHO: World Health Organization.

^f^ADPSC: Australasian Diabetes in Pregnancy Society criteria.

#### Secondary Outcomes: Maternal and Neonatal Outcomes

##### Effect on GWG

**Figure 4:**
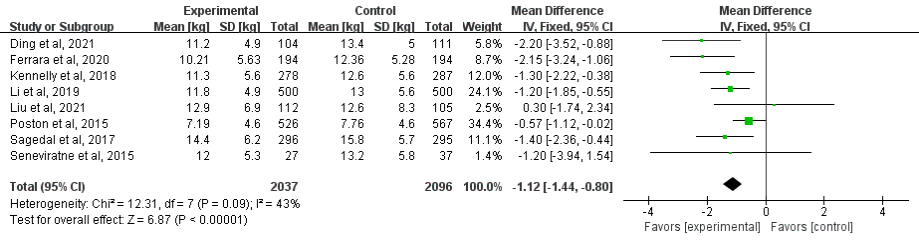
Forest plot of the effect of mobile health interventions on gestational weight gain [23, 29-31,34-37]. IV: inverse variance.

A total of 50% (8/16) of the studies examined the effects of mHealth-based interventions on GWG [23,29-31,34-37]. There were statistically significant differences in decreases in GWG between the mHealth intervention groups and the control groups (mean difference=−1.12 kg, 95% CI −1.44 to −0.80; *P<*.001; *I*^2^=43%; Figure 4 [23, 29-31,34-37]). The effects of the mHealth-based lifestyle interventions on maternal and neonatal outcomes are shown in Table 3.

**Table 3:** Effectiveness of mobile health–based lifestyle interventions on maternal and neonatal outcomes.

Maternal and neonatal outcomes			References		Studies, n (%)		Sample size		Statistical method		Effect estimates (95% CI)		*P* value
**Maternal outcomes**													
	Postpartum hemorrhage (%)	Wu and Guang [26] Ding et al [29] Dodd et all [32] Seneviratne et al [34] Poston et al [35] Sagedal et al [37]		6 (38)		4664		Odds ratio (IV^a^; fixed)		1.06 (0.90 to 1.24)		.49	
	Cesarean delivery (%)	Chen [22] Tang et al [24] Cao [25] Wu and Guang [26] Ferrara et al [30] Dodd et al [32] Seneviratne et al [34] Poston et al [35] Kennelly et al [36]		9 (56)		5224		Odds ratio (IV; fixed)		0.89 (0.79 to 1.00)		.05	
	Preeclampsia or PIH^b^ (%)	Wu and Guang [26] Ding et al [29] Ferrara et al [30] Liu et al [31] Dodd et al [32] Seneviratne et al [34] Poston et al [35] Kennelly et al [36] Sagedal et al [37]		9 (56)		5829		Odds ratio (IV; fixed)		0.96 (0.80 to 1.15)		.63	
	GWG^c^ (kg)	Li [23] Ding et al [29] Ferrara et al [30] Liu et al [31] Seneviratne et al [34] Poston et al [35] Kennelly et al [36] Sagedal et al [37]		8 (50)		4133		Mean difference (IV; fixed)		−1.12 (−1.44 to −0.80)		<.001	
**Neonatal outcomes**													
	Preterm birth (%)	Wu and Guang [26] Zhou et al [27] Kang and Sung [28] Ding et al [29] Liu et al [31] Dodd et al [32] Seneviratne et al [34]		7 (44)		2998		Odds ratio (IV; fixed)		0.65 (0.48 to 0.87)		.004	
	Macrosomia (%)	Li [23] Tang et al [24] Wu and Guang [26] Zhou et al [27] Kang and Sung [28] Ding et al [29] Ferrara et al [30] Liu et al [31] Dodd et al [32]		9 (56)		4449		Odds ratio (IV; random)		0.59 (0.40 to 0.87)		.008	
	LGA^d^ >90th percentile (%)	Dodd et al [32] Simmons et al [33] Poston et al [35] Kennelly et al [36] Sagedal et al [37]		5 (31)		4966		Odds ratio (IV; random)		0.80 (0.60 to 1.06)		.12	
	SGA^e^ <10th percentile (%)	Liu et al [31] Simmons et al [33] Kennelly et al [36] Sagedal et al [37]		4 (25)		1529		Odds ratio (IV; fixed)		1.10 (0.78 to 1.55)		.60	

^a^IV: inverse variance.

^b^PIH: pregnancy-induced hypertension.

^c^GWG: gestational weight gain.

^d^LGA: large for gestational age.

^e^SGA: small for gestational age.

##### Effect on Cesarean Delivery

A total of 56% (9/16) of the studies explored the effects of mHealth-based interventions on cesarean delivery [22,24-26,30,32,34-36]. The pooled results showed no significant difference between the mHealth intervention and control groups (OR 0.89, 95% CI 0.79-1.00; *P=*.05; *I*^2^=39%). Forest plots of maternal and neonatal outcomes are shown in Multimedia Appendix 6 [22-37].

##### Effect on Pregnancy-Induced Hypertension

A total of 56% (9/16) of the studies assessed the effects of mHealth-based interventions on the risk of pregnancy-induced hypertension [26,29-32,34-37]. Compared with the control groups, the groups with mHealth-based interventions did not show statistically significant decreases in pregnancy-induced hypertension (OR 0.96, 95% CI 0.80-1.15; *P=*.63; *I*^2^=31%).

##### Effect on Postpartum Hemorrhage

A total of 38% (6/16) of the studies assessed the effects of mHealth-based lifestyle interventions on the risk of postpartum hemorrhage [26,29,32,34,35,37]. There were no statistically significant decreases in postpartum hemorrhage between the groups with mHealth-based interventions and the control groups (OR 1.06, 95% CI 0.90-1.24; *P=*.49; *I*^2^=17%).

##### Effect on Preterm Birth

**Figure 5:**
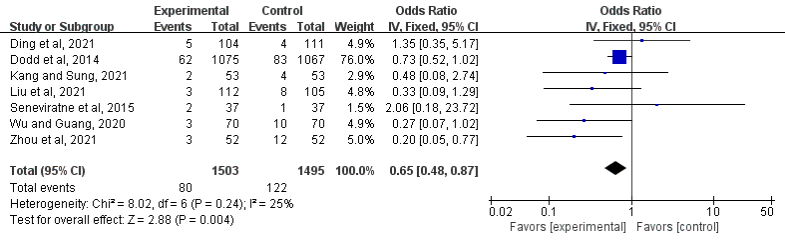
Forest plot of the effect of mobile health interventions on preterm birth [26-29,31,32,34]. IV: inverse variance.

A total of 44% (7/16) of the studies explored the effects of mHealth-based interventions on the risk of preterm birth [26-29,31,32,34]. Statistically significant differences in decreases in preterm birth were found between the mHealth intervention groups and the control groups (OR 0.65, 95% CI 0.48-0.87; *P*=.004; *I*^2^=25%; Figure 5 [26-29,31,32,34]).

##### Effect on Macrosomia

**Figure 6:**
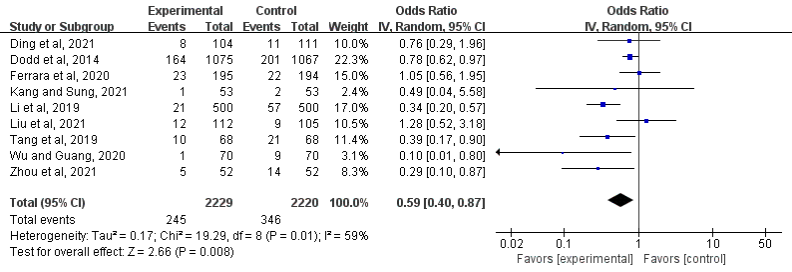
Forest plot of the effect of mobile health interventions on macrosomia [23, 24,26-32]. IV: inverse variance.

A total of 56% (9/16) of the studies evaluated the effects of mHealth-based interventions on the risk of macrosomia [23,24,26-32]. Statistically significant differences in decreases in macrosomia were found between the mHealth intervention groups and the control groups (OR 0.59, 95% CI 0.40-0.87; *P*=.008; *I*^2^=59%; Figure 6 [23,24,26-32]).

##### Effect on Large-for-Gestational-Age Infants

A total of 31% (5/16) of the studies explored the effects of mHealth-based lifestyle interventions on large-for-gestational-age infants [32,33,35-37]. Compared with the control groups, the mHealth intervention groups did not show statistically significant decreases in large-for-gestational-age infants (OR 0.80, 95% CI 0.60-1.06; *P*=.12; *I*^2^=51%).

##### Effect on Small-for-Gestational-Age Infants

In total, 25% (4/16) of the studies assessed the effects of mHealth-based lifestyle interventions on small-for-gestational-age infants [31,33,36,37]. Compared with the control groups, the mHealth intervention groups did not show statistically significant decreases in small-for-gestational-age infants (OR 1.10, 95% CI 0.78-1.55; *P*=.60; *I*^2^=0%).

## Discussion

### Methodological Quality of the Included Studies

The methodological quality of the included studies was assessed using the Cochrane risk-of-bias tool. A total of 25% (4/16) of the studies did not report details of randomization methods, and 75% (12/16) of the studies did not report allocation concealment and were at risk of selection bias. In total, 25% (4/16) of the studies did not blind participants and researchers. Therefore, there was a high risk of performance bias in these studies. A total of 12% (2/16) of the studies did not blind the outcome assessors and were rated as having a high risk of detection bias. The included studies reported complete data with low attrition bias. Finally, the funnel plot showed no substantial publication bias among the included studies. Therefore, the overall methodological quality was moderate, and larger samples and well-designed randomized trials are needed in the future.

### Summary of Principal Findings

#### Effectiveness of mHealth Interventions

A total of 16 RCTs were included in this systematic review and meta-analysis. All of these studies offered mHealth lifestyle interventions for perinatal women with overweight or obesity. Pooled results showed that the mHealth interventions reduced the incidence of GDM, which was consistent with the results of a previous study [38]. Various strategies have been proposed to prevent GDM effectively, and the primary intervention strategy is to change the lifestyle. Lifestyle interventions, including dietary guidance, physical exercise, weight management, health education, and blood glucose self-monitoring, are first-line strategies for GDM prevention [39]. The benefits of lifestyle interventions are mediated by mechanisms that improve glycemic variables and outcomes in type 4 and type 2 diabetes by increasing insulin sensitivity and reducing oxidative stress, which has been demonstrated in studies in other populations [40]. Compared with conventional lifestyle interventions, mHealth-based lifestyle interventions can make health education more attractive by providing more intuitive and vivid education and consultation with the help of electronic devices. In addition, pregnant women need to maintain close and continuous contact with the medical team during pregnancy and communicate effectively in the event of pregnancy complications and conscious fetal abnormalities. The advantage of mHealth technology is its ability to provide time-sensitive connectivity and high-quality health care for pregnant women in all regions [41]. mHealth lifestyle interventions enable pregnant women to maintain a healthy diet and engage in appropriate physical activity on a daily basis to encourage a healthier lifestyle during pregnancy, thus reducing the incidence of GDM.

#### Subgroup Analysis

In this review, we classified mHealth lifestyle interventions into 3 groups based on delivery approach: app-based interventions, phone-based interventions, and mHealth interventions delivered via a computer. Subgroup analysis demonstrated that app-delivered mHealth interventions were highly effective in reducing the risk of GDM in women with overweight or obesity. mHealth telephone-based interventions and interventions delivered via a computer had no significant effect on the prevention of GDM. Perinatal women usually have different problems and specific needs during pregnancy. Personalized applications can focus on individual characteristics and tailor goals and actions to diverse populations [42]. In this way, medical staff can leverage app-based mHealth interventions to provide health care tailored to the specific needs of pregnant women with overweight or obesity. In contrast, the applications help medical staff remotely monitor real-time parameters related to the health of pregnant women who are overweight or obese during pregnancy, observe whether they are adhering to a healthy and appropriate lifestyle, facilitate communication with them, and help them control their blood sugar and reduce the incidence of GDM [43].

Regarding the different providers of the interventions, the results of the subgroup analysis reported that mHealth interventions delivered by medical staff, including obstetricians and nurses, were effective in preventing GDM. It has been suggested that different models of care provided by various intervention providers may influence outcomes. mHealth interventions delivered by dietitians may focus on the food needs of women during pregnancy [44], whereas exercise physiologists are more concerned with physical activity during pregnancy [45]. Medical staff usually focus on the overall health of pregnant women, providing nutrition, exercise, weight management, and other comprehensive knowledge of pregnancy health care [46].

Subgroup analysis showed that mHealth interventions combining diet and exercise were effective in preventing gestational diabetes in women with overweight or obesity. Combined interventions with diet and exercise appeared to have a greater impact on GDM than interventions with diet or exercise alone, and the effects of diet and exercise on GDM were indistinguishable [47]. Type 2 diabetes has been proven to be preventable through combined diet, exercise, and weight loss interventions [48]. Another subgroup analysis found that mHealth-based lifestyle interventions were effective in preventing GDM in Asian populations. However, the pooled effects of interventions on the risk of GDM in predominantly White populations did not reach statistical significance. The disease burden of GDM varies by race because of multiple factors, including socioeconomic status, lifestyle, and culture, and the prevalence of GDM is significantly higher among Asian and Hispanic populations than among White populations [49]. The results of the subgroup analysis showed that, in studies using the IADPSG diagnostic criteria for GDM, mHealth-based lifestyle interventions had a significantly greater effect on GDM. The prevalence of GDM varies widely worldwide, at least in part because of a lack of consistency in screening and diagnostic criteria. Using lower glucose level thresholds as recommended by the IADPSG, significantly higher numbers of women with GDM were identified compared with using other diagnostic criteria [50].

#### Maternal and Neonatal Outcomes

Our meta-analyses revealed that the combined effect of mHealth interventions reduced the incidence of preterm birth, macrosomia, and excessive GWG. A review summarized the evidence regarding the influence of maternal diet before and during pregnancy on preterm birth. The results indicated that better maternal diet quality during pregnancy, characterized by a high intake of vegetables, fruits, whole grains, dairy products, and protein, played a significant role in reducing the risk of premature birth [51]. In addition, this study was in line with the findings of Fair and Soltani [52], who conducted a systematic review of the effectiveness of lifestyle interventions on weight gain in women with overweight or obesity during pregnancy and found that lifestyle interventions slightly reduced weight gain during pregnancy by 0.3 to 2.4 kg compared with standard care. There is no robust evidence that mHealth-based interventions are associated with a lower prevalence of postpartum hemorrhage, pregnancy-induced hypertension, cesarean sections, or any alteration in gestational age, consistent with a previous systematic review [53]. One reason for this nonsignificant effect may be the insufficient power of the combined sample size. Another possibility is the short duration of the interventions (median 18 wk), which might have been inadequate to affect some obstetric complications and neonatal outcomes. Further studies are needed to actively explore the associations between mHealth interventions and maternal and neonatal outcomes.

### Strengths and Limitations

This systematic review and meta-analysis has several strengths. We combined MeSH terms and keywords covering pregnancy and mHealth to conduct a comprehensive search in 5 primary English electronic databases and 4 main Chinese electronic databases to minimize the possibility of publication bias. We used a robust 3-step search strategy to include databases containing published and unpublished RCTs. To minimize bias, the review methods were preregistered in accordance with the PRISMA statement. In addition, some of the studies included in this meta-analysis were conducted in Western, high-income countries, and some were conducted in Eastern, lower-income countries. Our meta-analyses provided an excellent synthesis of the responses of participants from different cultural backgrounds to mHealth interventions.

However, there were also some limitations to this review. First, we only retrieved studies published in English or Chinese owing to language limitations. Studies published in other languages were not included in the review, which may have led to some publication bias. Second, the methodological quality of the included studies was not optimal, and some studies had a risk of performance bias and detection bias. Third, the included studies varied in sample size, participant characteristics, components of the mHealth interventions, and intervention implementation methods, which may have led to high heterogeneity. Moreover, key variables such as the exact start of the intervention and intensity of physical activity were missing and incomplete in some studies and may have biased the pooled effects. Finally, not all the included studies reported the safety and cost-effectiveness of the mHealth interventions.

### Conclusions

This systematic review and meta-analysis demonstrated that mHealth-based lifestyle interventions have a favorable impact on the prevention of GDM in pregnant women with overweight and obesity. mHealth interventions are a convenient and effective way to support pregnant women with overweight and obesity in out-of-hospital self-management in the context of rapid advances in IT and faster transmission speed. However, the potential of mHealth-based interventions for GDM needs to be further explored with better design and more rigorous large-scale RCTs.

## Appendix

Multimedia Appendix 1 PRISMA (Preferred Reporting Items for Systematic Reviews and Meta-Analyses) checklist.

Multimedia Appendix 2 Search strategies.

Multimedia Appendix 3 The details of the mobile health interventions.

Multimedia Appendix 4 Funnel plot.

Multimedia Appendix 5 Forest plots of subgroup analysis.

Multimedia Appendix 6 Forest plots of secondary outcomes.

## Abbreviations

GDM: gestational diabetes mellitus
GWG: gestational weight gain
IADPSG: International Association of Diabetes and Pregnancy Study Groups
MeSH: Medical Subject Headings
mHealth: mobile health
OR: odds ratio
PRISMA: Preferred Reporting Items for Systematic Reviews and Meta-Analyses
RCT: randomized controlled trial
