# Supplementary material for: Effects of mHealth-Based Lifestyle Interventions on Gestational Diabetes Mellitus in Pregnant Women With Overweight and Obesity: Systematic Review and Meta-Analysis
Source: JMIR Mhealth Uhealth. 2024 Jan 17;12:e49373. doi: 10.2196/49373 (PMC10831670; doi:10.2196/49373)
Supplement: Multimedia Appendix 2 [file mhealth_v12i1e49373_app2.docx]

# Supplementary Material 2. Search strategy for each database

**MEDLINE**

1. overweight[MeSH] OR obesity[MeSH] OR obes*[Title/Abstract] OR fat[Title/Abstract] OR unhealthy weight[Title/Abstract] OR high BMI[Title/Abstract] OR weight gain[Title/Abstract]
2. pregnancy[MeSH] OR pregnan*[Title/Abstract] OR prenatal*[Title/Abstract] OR antenatal*[Title/Abstract] OR maternal*[Title/Abstract] OR gestat*[Title/Abstract] OR diabetes, gestational[MeSH] OR gestational diabetes[Title/Abstract] OR gestational diabetes mellitus[Title/Abstract] OR GDM[Title/Abstract]
3. telemedicine[MeSH] OR Telehealth[Title/Abstract] OR telerehabilitation[MeSH] OR ehealth[Title/Abstract] OR mhealth[Title/Abstract] OR mobile health[Title/Abstract] OR telecare[Title/Abstract] OR telehealthcare[Title/Abstract] OR mcare[Title/Abstract] OR telemonitor*[Title/Abstract] OR telerehab*[Title/Abstract] OR telecommunications[MeSH] OR telemanagement[Title/Abstract] OR mobile communication[Title/Abstract] OR remote consult[Title/Abstract] OR mobile technolog*[Title/Abstract] OR mobile devic*[Title/Abstract] OR mobile app*[Title/Abstract] OR internet[MeSH] OR web*[Title/Abstract] OR online[Title/Abstract] OR smartphone[MeSH] OR mobile phone[Title/Abstract] OR telephone[MeSH] OR cell phone[MeSH] OR cellular phone[MeSH] OR messag*[Title/Abstract]
4. controlled clinical trial[Publication Type] OR randomized controlled trial[Publication Type] OR randomized[Title/Abstract] OR placebo[Title/Abstract] OR drug therapy[MeSH Subheading] OR randomly[Title/Abstract] OR trial[Title/Abstract] OR groups[Title/Abstract]) NOT (animals[MeSH] NOT humans[MeSH])
5. 1 and 2 and 3 and 4

**Embase(Ovid)**

1. exp Obesity/
2. (overweight or obes* or fat or unhealthy weight or high BMI or weight gain).ti,ab.
3. exp pregnancy diabetes mellitus/or (gestational diabetes or gestational diabetes mellitus or GDM).ti,ab.
4. exp Pregnancy/ or (pregnan* or prenatal* or antenatal* or maternal* or gestat*).ti,ab.
5. 1 or 2
6. 3 or 4
7. exp Telemedicine/
8. telerehabilitation/or telecommunications/or (telehealth or ehealth or mhealth or mobile health or telecare or telehealthcare or mcareor telemonitor* or telerehab* or telemanagement).ti,ab.
9. internet/ or smartphone/ or telephone/ or cellular phone/ or (cell phone or mobile communication or remote consult or mobile technolog* or mobile devic* or mobile app* or web* or online or mobile phone or messag*).ti,ab.
10. 7 or 8 or 9
11. randomized controlled trial/ or single blind procedure/ or crossover procedure/ or double blind procedure/ or (random* or factorial* or crossover* or cross over* or cross-over* or placebo* ).ti,ab.
12. (assign* or allocat* or volunteer*).ti,ab.
13. (animal/ or nonhuman/) not human/
14. (11 or 12 ) not 13
15. 5 and 6 and 10 and 14

**Cochrane Central Register of Controlled Trials (Cochrane Library)**

1. MeSH descriptor: [Overweight] explode all trees
2. MeSH descriptor: [Obesity] explode all trees
3. (obes* or fat or unhealthy weight or high BMI or weight gain):ti,ab,kw
4. 1 or 2 or 3
5. MeSH descriptor: [Diabetes, Gestational] explode all trees
6. (gestational diabetes or gestational diabetes mellitus or GDM):ti,ab,kw
7. MeSH descriptor: [Pregnancy] explode all trees
8. (pregnan* or prenatal* or antenatal* or maternal* or gestat*):ti,ab,kw
9. 5 or 6 or 7 or 8
10. MeSH descriptor: [Telemedicine] explode all trees
11. MeSH descriptor: [Telecommunications] this term only
12. (telerehabilitation or telehealth or ehealth or mhealth or mobile health or telecare or telehealthcare or mcareor telemonitor* or telerehab* or telemanagement):ti,ab,kw
13. MeSH descriptor: [Internet] this term only
14. MeSH descriptor: [Telephone] this term only
15. MeSH descriptor: [Cellular phone] this term only
16. (smartphone or cell Phone or mobile communication or remote consult or mobile technolog* or mobile devic* or mobile app* or web* or online or mobile phone or messag*):ti,ab,kw
17. 10 or 11 or 12 or 13 or 14 or 15 or 16
18. 4 and 9 and 17

**Web of Science**

1. TS =(Overweight OR Obesity OR Obes* OR Fat OR unhealthy weight OR high BMI OR Weight Gain)
2. TS =(Gestational diabetes or gestational diabetes mellitus or GDM or Pregnancy OR Pregnan* OR Prenatal* OR Antenatal* OR Maternal* OR Gestat*)
3. TS =(Telemedicine OR Telecommunications OR Telerehabilitation OR Telehealth OR Ehealth OR Mhealth OR mobile health OR Telecare OR Telehealthcare OR mcareor telemonitor* OR Telerehab* OR telemanagement OR Internet OR Telephone OR Cellular phone OR Smartphone OR cell Phone OR mobile communication OR remote consult OR mobile technolog* OR mobile devic* OR mobile app* OR Web* OR Online OR mobile phone OR Messag*)
4. TS =(placebo* or random* or clinical trial* or double blind* or single blind* or rct)
5. 1 and 2 and 3 and 4

**CINAHL (EBSCO)**

1. (MH "Overweight") OR (MH "Obesity")
2. (obes* or fat or unhealthy weight or high BMI or weight gain)
3. (MH "Gestational diabetes") OR (MH "Pregnancy")
4. (pregnan* or prenatal* or antenatal* or maternal* or gestat* or gestational diabetes mellitus or GDM)
5. 1 or 2
6. 3 or 4
7. (MH "Telemedicine") OR (MH“telerehabilitation”) OR (MH“telecommunications”)
8. TI (telehealth or ehealth or mhealth or mobile health or telecare or telehealthcare or mcareor telemonitor* or telerehab* or telemanagement)
9. (MH "Internet") OR (MH “Smartphone”) OR (MH”Telephone”) OR (MH”Cell phone”) OR (MH”Cellular phone”)
10. TI (mobile communication or remote consult or mobile technolog* or mobile devic* or mobile app* or web* or online or mobile phone or messag*)
11. 7 or 8 or 9 or 10
12. (MH "Clinical Trials") OR PT "Clinical Trial" OR (MH "Placebos") OR TX (placebo* OR assign* OR control* OR allocat* OR random*)
13. 5 and 6 and 11 and 12

**China National Knowledge Infrastructure (CNKI)**

1. （主题=超重）OR（主题=肥胖）
2. （主题=妊娠期糖尿病）OR（主题=妊娠糖尿病) OR（主题=妊娠合并糖尿病）
3. （主题=远程医疗）OR（主题=远程保健）OR（主题=手机）OR（主题=电脑）OR（主题=短信）OR（主题=微信）OR（主题=APP）
4. （摘要=随机对照试验）OR（摘要=随机对照）
5. 1 and 2 and 3 and 4

**Wanfang Database**

1. 主题:(超重) OR主题:(肥胖)
2. 主题:(妊娠期糖尿病) OR主题:(妊娠糖尿病) OR主题:(妊娠合并糖尿病)
3. 主题:(远程医疗) OR 主题:(远程保健) OR主题:(手机) OR 主题:(电脑) OR 主题:(短信) OR 主题:(微信) OR 主题:(APP)
4. 摘要:(随机对照试验) OR 摘要:(随机对照)
5. 1 and 2 and 3 and 4

**VIP Database**

1. 题名或关键词=超重 OR 题名或关键词=肥胖
2. 题名或关键词=妊娠期糖尿病 OR 题名或关键词=妊娠糖尿病 OR 题名或关键词=妊娠合并糖尿病
3. 题名或关键词=远程医疗 OR 题名或关键词=远程保健 OR 题名或关键词=手机 OR 题名或关键词=电脑 OR 题名或关键词=微信 OR 题名或关键词=APP
4. 摘要=随机对照试验 OR摘要=随机对照
5. 1 and 2 and 3 and 4

**Chinese Biomedical Literature Database**

1. "超重"[常用字段] OR "肥胖"[常用字段]
2. "妊娠期糖尿病"[常用字段] OR "妊娠糖尿病"[常用字段] OR "妊娠合并糖尿病"[常用字段]
3. "远程医疗"[常用字段] OR "远程保健"[常用字段] OR "手机"[常用字段] OR "电脑"[常用字段] OR "短信"[常用字段:] OR "微信"[常用字段] OR "APP"[常用字段] OR "应用"[常用字段])
4. "随机对照试验"[常用字段] OR "随机对照"[常用字段]
5. 1 and 2 and 3 and 4
