# Supplementary material for: Effects of mHealth-Based Lifestyle Interventions on Gestational Diabetes Mellitus in Pregnant Women With Overweight and Obesity: Systematic Review and Meta-Analysis
Source: JMIR Mhealth Uhealth. 2024 Jan 17;12:e49373. doi: 10.2196/49373 (PMC10831670; doi:10.2196/49373)
Supplement: Multimedia Appendix 3 [file mhealth_v12i1e49373_app3.docx]

**Supplementary Material 3. The details of the mHealth interventions.**

| Author, year, country | Type of intervention | Detail of intervention | Delivery mode | Duration | Provider | Theory-based |
| --- | --- | --- | --- | --- | --- | --- |
| Dodd et al.,2014, Australia | Diet+Exercise | (1)Provide dietary and physical activity advice. Participants were provided with dietary advice consistent with Australian standards to maintain a balance of carbohydrates, fat, and protein. Physical activity advice primarily encouraged participants to increase the amount of walking and incidental activity.  (2)Participants were provided with individualised information, including meal plans, healthy recipes, simple food substitutions, options for healthy snacking and eating out, and guidelines for healthy food preparation. Dietician (at 28 weeks’ gestation) and research assistants (via telephone call at 22, 24, and 32 weeks’ gestation and a face to face visit at 36 weeks’ gestation) monitored the progress of the participants. | Phone counselling | 16weeks | Dietician | Health decision making theory |
| Sagedal et al., 2015, Norway | Diet+Exercise | (1)The dietary component consisted of ten recommendations designed to increase awareness of food choices, dietary counselling was performed by telephone, with an initial consultation and then a follow-up 4–6 weeks later, each of approximately 20 minutes.  (2)The physical activity component consisted of access to twice-weekly exercise classes. The intensity of the exercise was self-monitored using Borg’s scale for perceived exertion.  (3)Participants were informed of the recommended GWG based on pre-pregnancy BMI and current IOM guidelines. | Phone counselling+website | 16weeks | Dietician and exercise physiologist | No |
| Seneviratne et al., 2015, New Zealand | Exercise | (1)Participants received a written programme prescribing frequency and duration of weekly exercises utilising magnetic stationary bicycles and provided with heart rate monitors to wear during all cycling sessions.  (2)Participants were visited at home at the beginning of the intervention by an exercise physiologist, who was available for help with exercise-related problems. The number of sessions completed and duration and intensity of cycling undertaken were obtained by downloading heart rate monitor data. | Software+device | 16weeks | Dietician and exercise physiologist | No |
| Poston et al.,2015, UK | Diet+Exercise | (1)Participants attended eight further health trainer-led group or individual sessions of 1 h duration once a week for 8 weeks. If a participant could not attend a session in person, the material was covered by telephone or email.  (2)Participants received advice on: self-monitoring, identification, and problem-solving of barriers to behaviour change; enlisting social support; providing opportunities for social comparison and received a DVD of an exercise regimen that was safe for pregnancy, a pedometer, and a log book for recording weekly SMART goals. | Phone+email+DVD+pedometer+log book | 8 weeks | Nurse and obstetrician | Control theory and social cognitive theory |
| Simmons et al.,2017, Australia | Diet+Exercise | (1)The diet intervention(HE) promoted a food-based, lower simple and complex carbohydrate, lower fat, higher fiber, higher protein diet.  (2)The PA intervention promoted both aerobic and resistance physical activity. All interventions recommended a limitation in GWG to 5 kg. The messages were supported by a toolkit for each participant, including the participant handbook, educational materials.  (3)The number of contacts and time included 5 face-to-face sessions of approximately 30 to 45 minutes duration, and ≤ 4 telephone calls of ≤ 20 minutes or contacts using electronic mail. At least 4 face-to-face coaching sessions were expected to occur before the second measurement session (24 to 28 weeks), and the intervention was completed by 35 weeks of gestation. | Email+phone+pedometer+device | 18weeks | Nurse and obstetrician | Social cognitive theory |
| Chen, 2017, China | Diet+Exercise | (1)The Dietary intervention guide participants to choose reasonable food and record a food diary. Regular follow-up visits by telephone or wechat or QQ were conducted every week.  (2)The physical activity intervention guided participants to exercise at an appropriate time and intensity, with regular weekly follow-up via phone, wechat or QQ. The weight was measured and exercise was adjusted at every prenatal examination of 29 to 36 weeks of pregnancy, and the motor log was recorded every day to maintain continuous movement. | Mobile apps+SMS | 24weeks | Nurse and obstetrician | No |
| Kennelly et al.,2018, Ireland | Diet | (1)Participants received a Healthy Lifestyle Package, which began with a single face-to-face education session centered on targeted nutrition and physical activity advice. The information received at this education session was reinforced through the following delivery channels: a smartphone application, emails every 2 weeks, and two follow-up face-to-face hospital visits at 28 and 34 weeks of gestation.  (2)The smartphone application consisted of three components: a comprehensive database of low GI recipes, a homepage comprising daily nutritional and an encouraging thought of the day. | Mobile app+email | 24weeks | Nurse and obstetrician | Control theory and social cognitive theory |
| Li, 2018, China | Diet+Exercise | (1)At 13 to 28 weeks of gestation, the intervention was performed once a month, 20min each time, a total of 4 times, and the nutritionist was invited to formulate an individual diet plan for the participants. Individualized exercise programs were designed according to the daily amount of exercise and BMI of participants.  (2)Every week, the participants were followed up by telephone once a week for 5 to 15 minutes, including gestational weight gain, diet and exercise. | Phone counselling | 24weeks | Nurse and obstetrician | Social cognitive theory |
| Tang et al.,2019, China | Diet+Exercise | Participants used the designed perinatal nutrition software and the diet assessment wechat platform to guide pregnant women on their diet and activities once every four weeks from 20 to 24 weeks of pregnancy. | Mobile app+software | 28weeks | Nurse and obstetrician | No |
| Ferrara et al.,2020, USA | Exercise | (1)The intervention, included 13 weekly individual sessions. The first and last sessions were in person and the remaining 11 were delivered by telephone. At the initial in-person session participant were advised of their GWG goal for the end of pregnancy.  (2)Women were provided a printed workbook to discuss at each session; a scale to encourage self-weighing; and a personalised electronic or paper-based graph to track their weight. | Phone+device | 13 weeks | Nurse and obstetrician | Social cognitive theory and and transtheoretical model |
| Cao, 2020, China | Diet+Exercise | (1)The interventions included personalized eating regiments for participants and guidance on the intensity and duration of exercise.  (2)Follow-up was conducted weekly through QQ, wechat, telephone and other methods, and the first face-to-face intervention was conducted at 13 to 28 weeks of pregnancy to check the implementation of diet and exercise plan. Maternal weight was measured between 29 and 36 weeks during each prenatal visit. | Mobile apps+SMS | 28weeks | Nurse and obstetrician | No |
| Wu and Guang, 2020, China | Diet+Exercise | Participants were informed of their weight during pregnancy through wechat groups. Dietitians made dietary plans for the participants during pregnancy and clinic staff selected low to moderate intensity aerobic exercise according to the participants' conditions, and gave exercise guidance. | Mobile app | 24 weeks | Nurse and obstetrician | No |
| Liu et al.,2021, USA | Diet+Exercise | (1)Intervention included an in-depth counseling session (≤18 weeks’ gestation) and 10 weekly group sessions, at which the interventionist shared the participant’s printed report of her dietary intake and PA (based on the dietary recalls and objective assessment of PA) and a personalized weight-gain-tracking graph.  (2)Participants set a PA and diet goal. Participants also received a binder of study handouts, a pedometer, and a bathroom scale. | Phone+software+device+SMS | 10 weeks | Nurse and obstetrician | Social cognitive theory |
| Zhou et al.,2021, China | Diet | (1)Interventions include regular nutritional guidance for pregnant women, basic dietary information during pregnancy, standardized measurements of pregnant women's body mass, and assessment of body mass growth in compliance with standards.  (2)Participants were provided with nutritional status, mental and behavioral health guidance through wechat, telephone or family follow-up. | Mobile app | 4 weeks | Nurse and obstetrician | No |
| Kang and Sung, 2021, China | Diet | (1)Interventions include a program for dietary education and management on the wechat platform, and daily release of nutrition guidance for perinatal pregnant women.  (2)Participants can input their food into the program to get their main intake of nutrition and adjust their diet accordingly. The program will remind participants to control their daily food intake, as well as provide health education and dietary guidance. | Mobile app | 12 weeks | Nurse and obstetrician | No |
| Ding et al.,2021, China | Diet+Exercise | (1)Participants were enrolled in the wechat group where they may ask questions about diet at any time and get answers from a dietitian. Dieticians also send messages in this wechat group with reference to dietary guidelines for pregnant women at regular intervals (once a week).  (2)Ideal body weight was assessed and used for calculating energy requirement. In addition, participants were asked to initiate a daily exercise plan that is, taking a walk for at least 6000 steps per day. Daily walking data were obtained from participants’ smartphones. | Mobile app | 16 weeks | Dietitian | No |

Abbreviations: BMI= body mass index, GWG= gestational weight gain, IOM= Institute of Medicine, GI= Glycemic index, PA= physical activity, app= application, SMS=short message service.
