# Supplementary material for: Effects of mHealth-Based Lifestyle Interventions on Gestational Diabetes Mellitus in Pregnant Women With Overweight and Obesity: Systematic Review and Meta-Analysis
Source: JMIR Mhealth Uhealth. 2024 Jan 17;12:e49373. doi: 10.2196/49373 (PMC10831670; doi:10.2196/49373)
Supplement: Multimedia Appendix 4 [file mhealth_v12i1e49373_app4.docx]

# Supplementary Material 4. Funnel plot


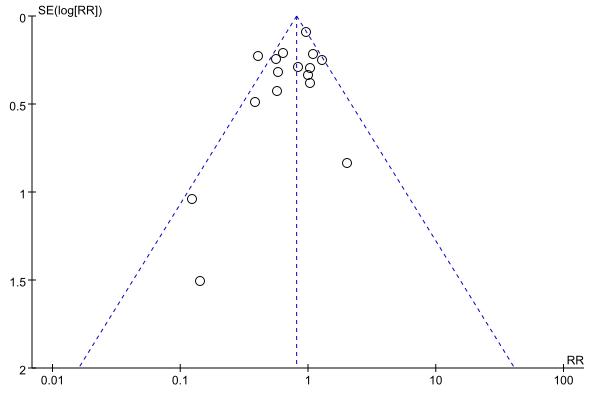


**Fig. 1** Funnel plot of publication bias. SE = standard error, RR = risk ratio.
