# Supplementary material for: Effects of mHealth-Based Lifestyle Interventions on Gestational Diabetes Mellitus in Pregnant Women With Overweight and Obesity: Systematic Review and Meta-Analysis
Source: JMIR Mhealth Uhealth. 2024 Jan 17;12:e49373. doi: 10.2196/49373 (PMC10831670; doi:10.2196/49373)
Supplement: Multimedia Appendix 5 [file mhealth_v12i1e49373_app5.docx]

# Supplementary Material 5. Forest plots of subgroup analysis


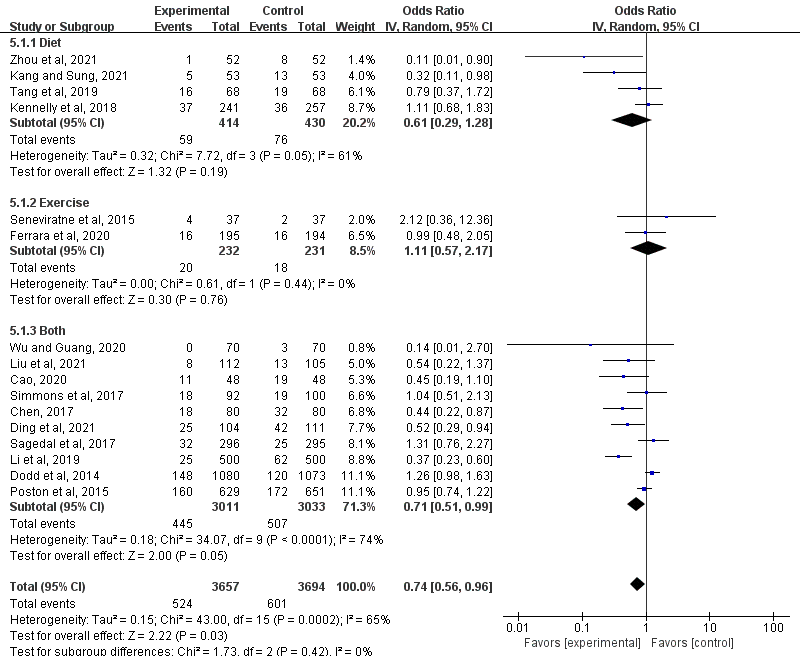


**Fig. 1.** Forest plot of effect of mHealth-based different interventions.



**
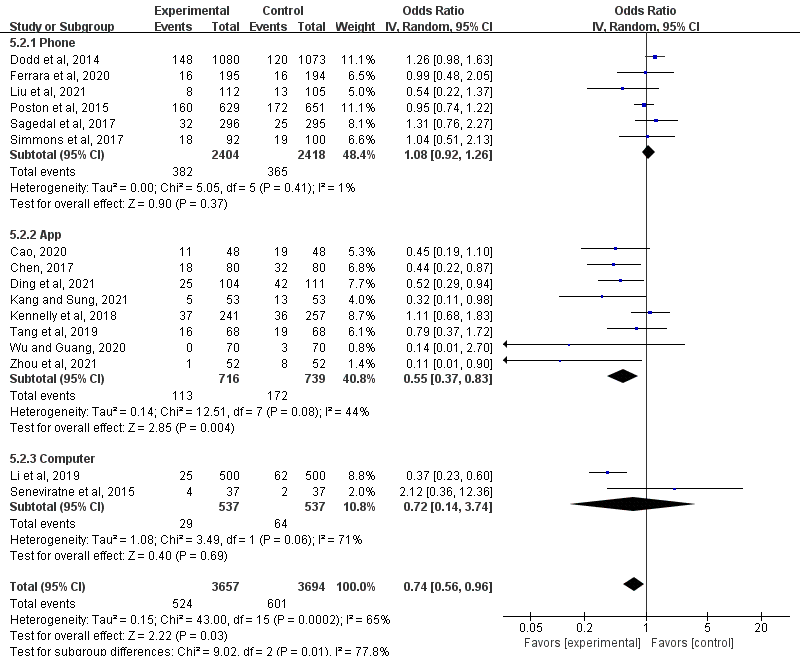
**

**Fig. 2.** Forest plot of effect of different mHealth technologies.

**
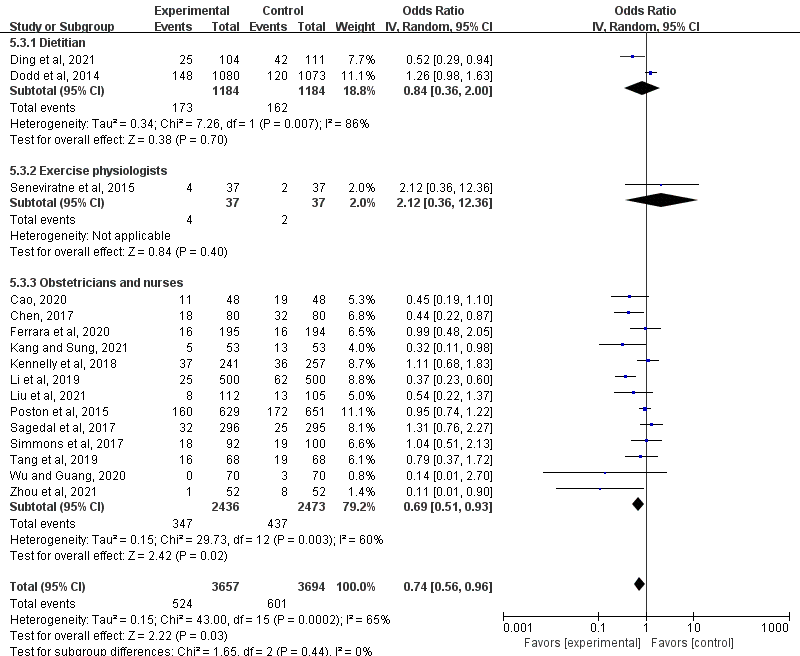
**

**Fig. 3.** Forest plot of effect of different providers.

**
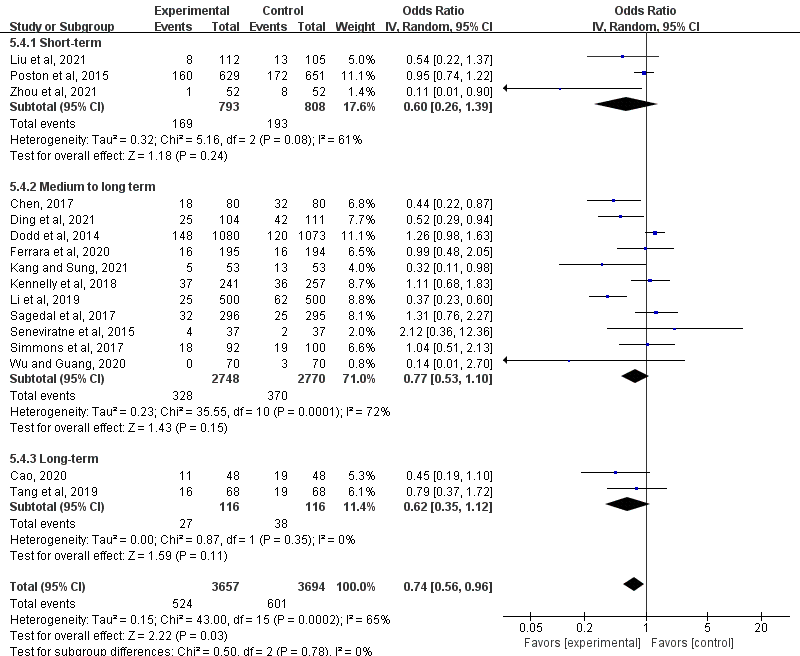
**

**Fig. 4.** Forest plot of effect of different durations.


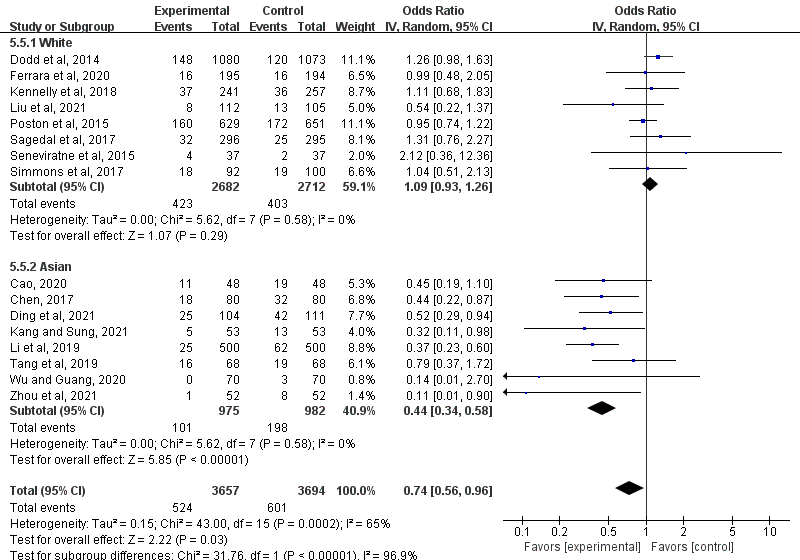


**Fig. 5.** Forest plot of effect of different ethnic groups.


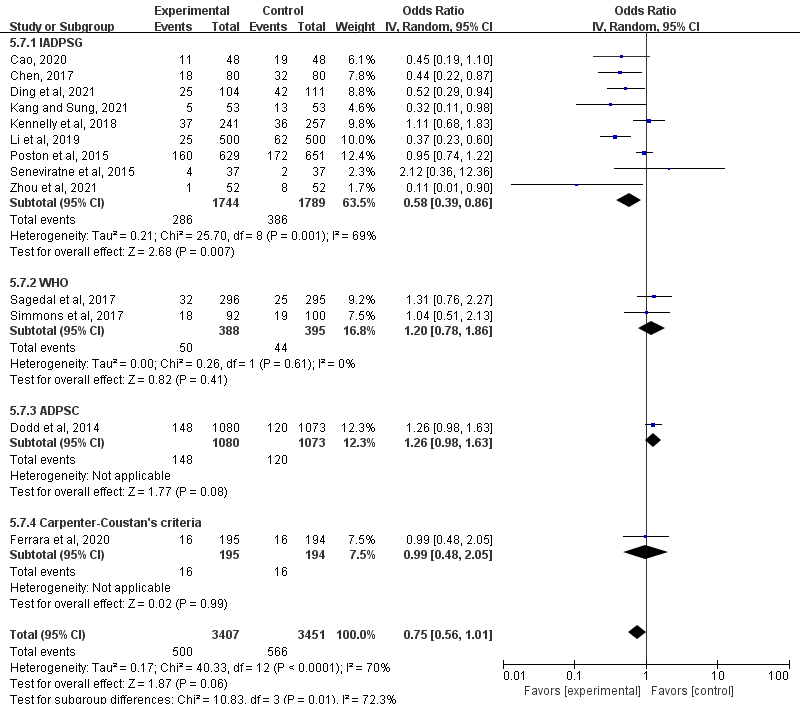


**Fig. 6.** Forest plot of effect of different GDM diagnostic criteria.
