# Supplementary material for: Effects of mHealth-Based Lifestyle Interventions on Gestational Diabetes Mellitus in Pregnant Women With Overweight and Obesity: Systematic Review and Meta-Analysis
Source: JMIR Mhealth Uhealth. 2024 Jan 17;12:e49373. doi: 10.2196/49373 (PMC10831670; doi:10.2196/49373)
Supplement: Multimedia Appendix 6 [file mhealth_v12i1e49373_app6.docx]

# Supplementary Material 6. Forest plots of secondary outcomes


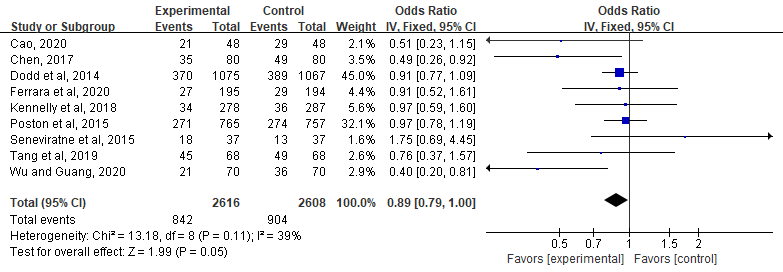


**Fig. 1.** Forest plot of caesarean delivery.


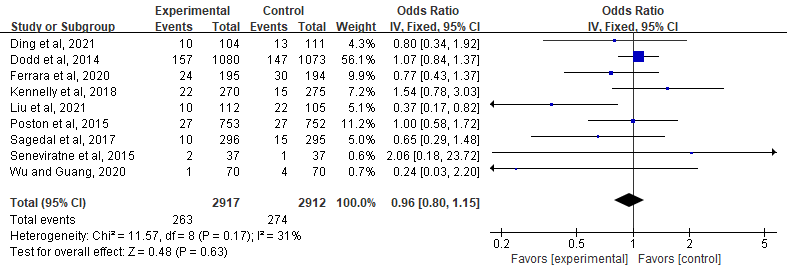


**Fig. 2.** Forest plot of pregnancy-induced hypertension.


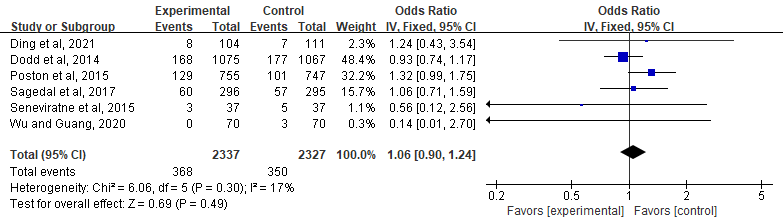


**Fig. 3.** Forest plot of postpartum hemorrhage.


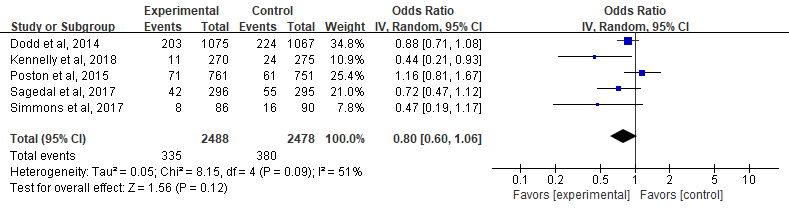


**Fig. 4.** Forest plot of large for gestational age infant.


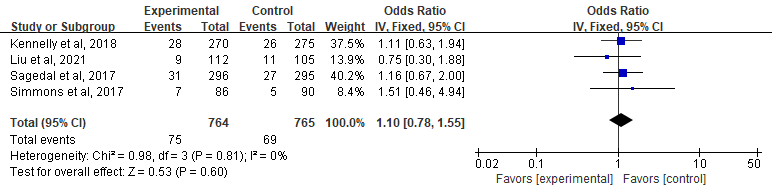


**Fig. 5.** Forest plot of small for gestational age infant.


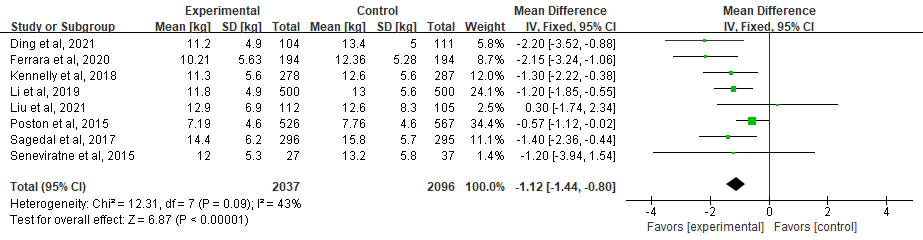


**Fig. 6.** Forest plot of gestational weight gain.


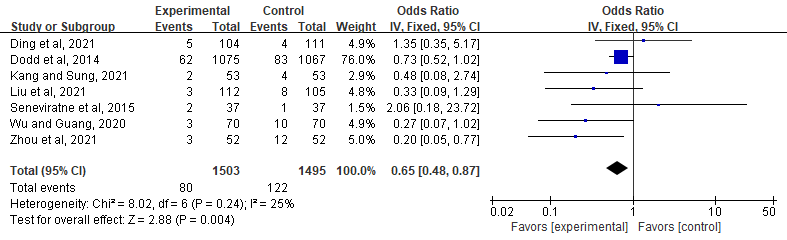


**Fig. 7.** Forest plot of preterm birth.


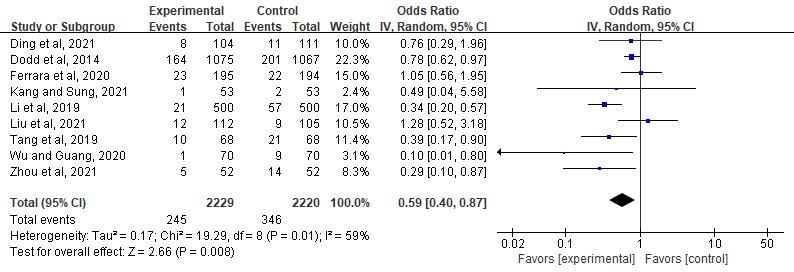


**Fig. 8.** Forest plot of macrosomia.
